# Engaging Moms on Teen Indoor Tanning Through Social Media: Protocol of a Randomized Controlled Trial

**DOI:** 10.2196/resprot.6624

**Published:** 2016-11-29

**Authors:** Sherry L Pagoto, Katie Baker, Julia Griffith, Jessica L Oleski, Ashley Palumbo, Barbara J Walkosz, Joel Hillhouse, Kimberly L Henry, David B Buller

**Affiliations:** ^1^ Division of Preventive and Behavioral Medicine Department of Medicine University of Massachusetts Medical School Worcester, MA United States; ^2^ Department of Community & Behavioral Health College of Public Health East Tennessee State University Johnson City, TN United States; ^3^ Klein Buendel, Inc. Golden, CO United States; ^4^ Department of Psychology Colorado State University Fort Collins, CO United States; ^5^ Colorado School of Public Health Colorado State University Fort Collins, CO United States

**Keywords:** skin cancer, indoor tanning, melanoma, Facebook, social media, health communication

## Abstract

**Background:**

Indoor tanning elevates the risk for melanoma, which is now the most common cancer in US women aged 25-29. Public policies restricting access to indoor tanning by minors to reduce melanoma morbidity and mortality in teens are emerging. In the United States, the most common policy restricting indoor tanning in minors involves parents providing either written or in person consent for the minor to purchase a tanning visit. The effectiveness of this policy relies on parents being properly educated about the harms of indoor tanning to their children.

**Objective:**

This randomized controlled trial will test the efficacy of a Facebook-delivered health communication intervention targeting mothers of teenage girls. The intervention will use health communication and behavioral modification strategies to reduce mothers’ permissiveness regarding their teenage daughters’ use of indoor tanning relative to an attention-control condition with the ultimate goal of reducing indoor tanning in both daughters and mothers.

**Methods:**

The study is a 12-month randomized controlled trial comparing 2 conditions: an attention control Facebook private group where content will be relevant to teen health with 25% focused on prescription drug abuse, a topic unrelated to tanning; and the intervention condition will enter participants into a Facebook private group where 25% of the teen health content will be focused on indoor tanning. A cohort of 2000 mother-teen daughter dyads will be recruited to participate in this study. Only mothers will participate in the Facebook groups. Both mothers and daughters will complete measures at baseline, end of intervention (1-year) and 6 months post-intervention. Primary outcomes include mothers’ permissiveness regarding their teenage daughters’ use of indoor tanning, teenage daughters’ perception of their mothers’ permissiveness, and indoor tanning by both mothers and daughters.

**Results:**

The first dyad was enrolled on March 31, 2016, and we anticipate completing this study by October 2019.

**Conclusions:**

This trial will deliver social media content grounded in theory and will test it in a randomized design with state-of-the-art measures. This will contribute much needed insights on how to employ social media for health behavior change and disease prevention both for indoor tanning and other health risk behaviors and inform future social media efforts by public health and health care organizations.

**ClinicalTrial:**

Clinicaltrials.gov NCT02835807; https://clinicaltrials.gov/ct2/show/NCT02835807 (Archived by WebCite at http://www.webcitation.org/6mDMICcCE).

## Introduction

### Indoor Tanning, Melanoma, and Public Policy

Indoor tanning elevates the risk for melanoma [1,2], which is now the most common cancer in women aged 25-29 [3,4]. Reducing indoor tanning by minors can prevent ultraviolet radiation exposure, a human carcinogen in the same class as arsenic and tobacco [2] and a primary risk factor for melanoma especially at young ages [5-11]. Indoor tanning before age 40 doubles the risk of melanoma; each tanning bed use per year increases risk for melanoma by 1.8% [12,13]. The increase in melanoma is especially evident in young, non-Hispanic, white women, paralleling the rise in their indoor tanning over the same period [14]. The Centers for Disease Control’s (CDC) Healthy People 2020 and Surgeon General’s Call to Action to Prevent Skin Cancer [15] have set the goal of reducing the prevalence of indoor tanning by teens. Currently, 10% to 15% of teens [16-20] (mainly girls) and 8% to 14% of caregivers [17,21-24] (mainly mothers) reported indoor tanning in the past year. Despite the substantial risk, indoor tanning remains popular among older adolescent females and mothers aged 27-45 [25].

Public policies restricting access to indoor tanning by minors to reduce melanoma morbidity and mortality in teens are emerging. Policy interventions can alter risk perceptions, preferences for risky behaviors, and barriers to change [26-28]. Currently, 29 states require parental permission for minors to indoor tan. Fewer states (n=24) have adopted indoor tanning regulations with age restrictions on access to tanning facilities, with just 13 states and 1 territory banning all minors under 18, making parental-permission regulations far more common than complete bans. Policies restricting minors’ access to indoor tanning will only reduce melanoma morbidity and mortality if the tanning industry complies with them [29,30]), states enforce them, and in the case of parental permission laws, parents withhold permission from teens who want to indoor tan. Unfortunately, research suggests that parental permission policies are not currently reducing rates of indoor tanning by minors [18] due to industry noncompliance, insufficient policy enforcement [29,30], and the fact that many parents fail to recognize the dangers of indoor tanning [22,31]. Exemplifying the latter, one study found 51% of mothers exhibited very little knowledge of the health consequences of indoor tanning [22]. Most (79%) also did not know that a “base tan” from a tanning bed is not protective and many (40%) were not aware that indoor tanning is potentially more harmful to teens than adults [31]. This lack of knowledge may be due to poor dissemination of information. The Food and Drug Administration provides some guidelines for exposure limits but it has only recently required facilities to post warnings on tanning beds [32-34].

Health communication that maximizes the effectiveness of indoor tanning policy, including both parental consent and bans, might activate mothers to protect their teen daughters from the harms. The current study fills this gap in the literature by testing a social media–delivered intervention developed to educate mothers and urge them to withhold permission for daughters to indoor tan. Mothers are an important target because their permissiveness and tanning behavior are strong predictors of daughters’ indoor tanning [18,22,31]. For instance, past-year indoor tanning rates are as high as 30% to 55% in teens whose mothers tan indoors [18,22,31]. Teen girls often report initiating indoor tanning with their mothers [35]. Further, girls whose first indoor tanning experience is with their mothers begin at an earlier age, become more habitual tanners, and are more resistant to change [35]. Recent research indicates that well-crafted communication can reduce maternal permissiveness about indoor tanning [36], but such communication has not been tested as a strategy specifically for maximizing indoor tanning policies. This trial leverages both health communication and behavioral strategies to improve the impact of public health policy. A social media campaign for mothers has the potential to reduce not only the prevalence of indoor tanning in adolescent girls but also the incidence of melanoma in young women. If effective, it could also easily and inexpensively be delivered by cancer-related public health organizations, many of which have social media feeds.

The present paper describes the design and methods of a randomized controlled trial of a Facebook-delivered health communication intervention to reduce mothers’ permissiveness regarding their teenage daughters’ use of indoor tanning, reduce their teenage daughters’ perception of their mothers’ permissiveness, and reduce indoor tanning by both mothers and daughters.

### Hypotheses

The primary hypothesis is that the intervention will significantly reduce mothers’ permissiveness regarding their daughters’ indoor tanning, their daughters’ perception of maternal permissiveness toward indoor tanning, and both mothers’ and daughters’ indoor tanning relative to the control condition. The secondary hypothesis is that a significantly greater number of mothers will support a ban on indoor tanning for minors in the intervention group compared with the control condition.

## Methods

### Pilot Data

Pilot interviews were conducted with 19 mothers of teenage daughters. Interviews included opinions of indoor tanning, indoor tanning policy, and health topic concerns as they relate to their daughters. Overall, 84% (16/19) were concerned about their daughters going indoor tanning, however 32% (6/19) would allow it. Most (16/19, 84%) would sign a petition supporting an indoor tanning ban for minors. Obesity and sexual activity were the greatest health concerns for daughters (both: 5/19, 31%), followed by drug and alcohol use (4/19, 26%), exercise (3/19, 16%), nutrition (3/19, 16%), mental health (3/19, 16%), cancer (2/19, 11%), and sleep (1/19, 5%). Most (15/19, 79%) mothers reported that they get health information from the Internet. This pilot study confirmed that many mothers would benefit from messages about the harms of allowing their daughters to indoor tan. It also helped us identify health topics of high interest to mothers.

### Study Design

**Figure 1:**
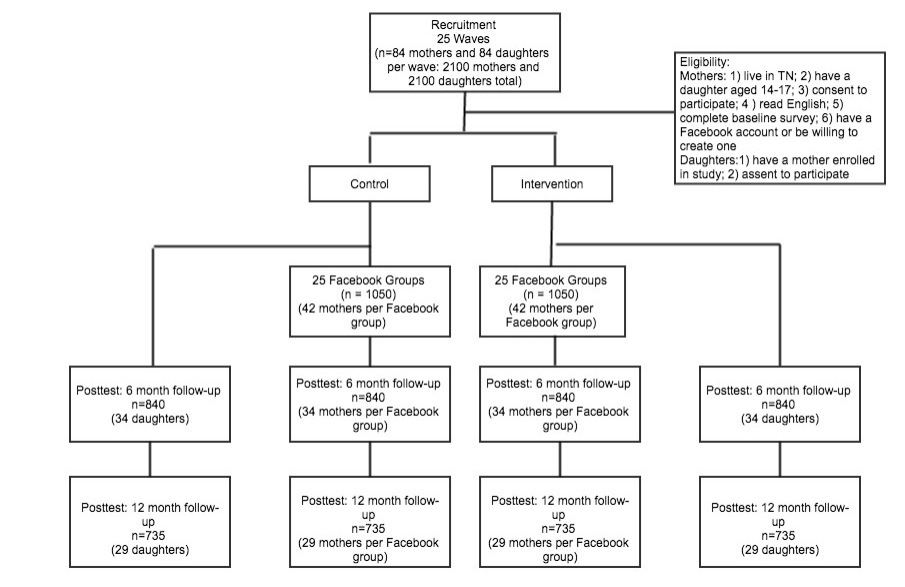
Study design.

The study design is a randomized controlled trial comparing 2 conditions over 1 year (Figure 1). Participants will be recruited in waves of approximately 84 and then randomized into the intervention or control condition. We continue in this way until a total of 25 waves have been enrolled, producing 25 groups in the intervention condition and 25 groups in the control condition each with approximately 42 participants. Participants will be blinded to condition and assessment points will occur at baseline, end of intervention (1-year), and 6 months postintervention.

### Intervention Condition

In the intervention condition, participants will enter a private Facebook group that posts a feed of health messages in which 25% are focused on preventing indoor tanning and 75% are focused on other health topics (eg, nutrition, physical activity, etc). Indoor tanning increases in December, peaking in March for seasonal tanners (eg, event and regular seasonal tanners) [37,38]. During these months, indoor tanning posts will be scheduled at a higher frequency (30% of posts).

### Control Condition

In the control condition, participants will enter a private Facebook group that posts a feed of health messages in which 25% are focused on preventing prescription drug abuse and 75% are focused on the same health topics as the intervention condition. We selected prescription drug abuse as our “control” content, because (1) it is completely unrelated to tanning, thus not likely to impact our primary outcomes, and (2) it is an emerging issue of great interest and relevance to young adults in east Tennessee. This 25% segment of posts is the only difference between the intervention and control conditions.

### Setting

The study is being conducted in east Tennessee given that Tennessee has a parental permission law for indoor tanning and a high prevalence of indoor tanning, with 31% of adolescent girls reporting indoor tanning in a recent study [39]). Tennessee’s indoor tanning policy (Tennessee Code Ann. § 68-117-104) requires that children under the age of 14 be accompanied by a parent if they use a commercial tanning facility and those ages 14 to 17 must have parents visit the tanning facility to sign a permission form in-person. The form only needs to be signed once at each facility. Communities in the region are diverse in size and rural/urban context and 84% of the public school population is white, the racial group most likely to indoor tan and at highest risk for melanoma [40].

### Participants

A cohort of 2000 mother-teen daughter dyads will be recruited to participate in this study. Only mothers and daughters will be recruited because female teens are nearly 4 times more likely to indoor tan (23% in 2009-2011 Youth Risk Behavior Survey) than male teens (6% [41]) and evidence suggests maternal permissiveness is a predictor of indoor tanning. The literature on indoor tanning by male teens is nascent with minimal data on predictors or effective intervention strategies.

Eligible mothers will meet the following criteria: (1) live in Tennessee, (2) have a daughter aged 14 to 17, (3) consent to participate, (4) read English, (5) complete the baseline survey, and (6) have a Facebook account (or be willing to create one). History of indoor tanning is not required for inclusion. Because public policy requires broad public support not just support by those most affected, ethnic minority mothers who are interested in participating (mainly African Americans; 14% are minority, 86% are non-Hispanic white) will be enrolled. The planned sample size was increased to ensure that statistical power is based on the number of non-Hispanic, white mothers. This approach will also allow for an evaluation of how the intervention affects ethnic minority mothers’ support for indoor tanning bans.

Eligibility criteria for teen daughters include having a mother enrolled in the study and assenting to participate. Daughters will be enrolled regardless of their indoor tanning behavior and the sample will be inflated to insure adequate numbers of non-Hispanic whites. Daughters will be enrolled only to complete assessments. They will not have access to the Facebook group. If a mother has more than one eligible daughter, she will provide information for the one with the nearest birthday, as instructed on the enrollment website.

### Sample Size and Power Calculations

Using public high school enrollment data, we estimate that approximately 20,000 eligible mothers and 25,000 eligible daughters reside in the east Tennessee region. Effect sizes for similar previous studies are in the range of moderate to large for our primary and secondary outcomes (mothers’ permissiveness and indoor tanning behavior, respectively). For example, Baker et al [42] found that mothers’ permissiveness and daughters’ perceptions of indoor tanning declined following a 1-month intervention at an immediate posttest (mother: baseline mean 2.59 [SD 1.03], follow-up mean 2.47 [SD 0.86]; daughter: baseline mean 3.12 [SD 1.32], follow-up mean 2.74 [SD 1.1]) compared with controls (mother: baseline mean 3.02 [SD 1.08], follow-up mean 2.98 [SD 1.10]; daughter: baseline mean 3.12 [SD 1.24], follow-up mean 3.40 [SD 1.04]). Likewise, for indoor tanning behavior, Hillhouse et al [43] found the number of sessions in the past 3 months among indoor tanners was reduced in an intervention condition at 6 months (baseline mean 4.67 [SD 0.60], follow-up mean 6.80 [SD 0.93]) compared with controls (baseline mean 4.48 [SD 0.55], follow-up mean 10.90 [SD 0.93]). The frequency of indoor tanning observed in the proposed study will likely be lower, because not all mothers and daughters will indoor tan (31% of adolescent girls indoor tanned in a recent study in east Tennessee; indoor tanning is higher in rural areas [44]). Still, the effect size between conditions is expected to remain moderate to large.

We used the Optimal Design software package (version 3.0) [45] to determine sample size. Assuming a 2-tailed alpha of 0.05, a moderate effect size of d=0.50, and an intraclass correlation of 0.05 within each Facebook private group, we far exceed a power of 0.80 with 50 Facebook private groups each consisting of 25 mothers (total n=1250 mothers and 1250 daughters). We increased this sample size to account for the proportion of minority mothers and daughters (15%) we expect to recruit to achieve the needed sample size of non-Hispanic whites, the racial/ethnic group most likely to indoor tan and with the highest rates of melanoma [40]. We further inflated the sample to account for an expected loss to follow-up of 30% by 12 months (20% at 6 months). Thus, we will recruit initial samples of 2100 mothers and 2100 daughters (42/Facebook group) at baseline and expect to successfully assess 1680 in each sample (approximately 34/Facebook group) at the 6-month follow-up and 1470 in each sample (approximately 29/Facebook group) at the 12-month follow-up, with the final samples containing 1250 non-Hispanic white mothers and 1250 non-Hispanic white daughters for analysis.

### Recruitment

Mother-daughter dyads will be recruited across 40 counties in east Tennessee using two primary strategies: (1) Coordinated School Health (CSH) Coordinators in each school system will provide access to mothers and daughters in high schools, and (2) study staff will recruit mothers and daughters through partnerships with community-based organizations (eg, churches, sports leagues, clubs, health clinics, etc). A local Expert Advisory Board, made up of regional CSH Coordinators, public health educators, and maternal and child health professionals is providing insight into effective community-based recruitment. CSH, which is housed in the Tennessee Department of Education, has the mission of working with schools and parents to improve children’s health, making them a natural partner in this effort. CSH Coordinators are asked to send study invitations to mothers through their normal channels (eg, back-to-school packets, flyers with report cards, email, newsletters, etc). Access to families through schools can be challenging, but partnering with CSH ensures we will not interfere with time, curricular, and other constraints. Schools that assist with mother-daughter recruitment will receive a US $200 mini-grant for CSH-related program materials. At the same time, study staff will systematically canvass communities across the region, beginning in the far northeast corner of the state and working their way south and west to partner with local organizations, media outlets, and employers to advertise the study to mothers and teen daughters in the region. We chose multiple recruitment methods based on our past experience recruiting women and adolescent girls in this hard-to-reach population [46]. CSH Coordinators and community-based organizations often have direct access to mothers of high school students and can serve as credible recruiters. For eligible participants, we expect a refusal rate of 30%, based on our previous experiences in this population [46].

Mothers are the target of recruitment efforts and must enroll in the trial first and then provide permission for their daughters to participate along with their daughters’ contact information. Interested mothers will sign up for the trial by visiting a study website where information is provided along with a screener that asks if they have a daughter ages 14-17 in the home, if they are a Tennessee resident, and if they have or are willing to have a Facebook account. Eligible mothers are sent to the consent and baseline survey.

When a mother completes the baseline survey, the enrollment website will send invitations to the daughter to assent and complete her own baseline survey. These invitations will be sent by email with up to 5 weekly reminders. Mothers remain eligible and enrolled in the study even if daughters do not provide assent; in such cases, daughters’ missing responses will be imputed.

### Intervention

#### Conceptual Framework

The intervention, named *Health Chat*, will be delivered in a private Facebook group. It was designed using an integrated conceptual framework combining 3 complementary theories of social and individual change to guide the intervention social media posts and attempts to generate user engagement in the Facebook groups. Content of the social media posts from the social media intervention were designed based on principles of social cognitive theory (SCT) [47,48] and transportation theory (TT) [49,50]. From SCT, the posts were written to address the social situation (increasing perceived social norms to not indoor tan or give permission for daughter to tan), behavioral capability (knowledge of the risks of indoor tanning and skills to refuse indoor tanning requests and invitations), expectations (belief that indoor tanning increases risk for melanoma), observational learning (in stories from real mom’s about the dangers of letting their daughters indoor tan, including about daughters who developed melanoma as young adults), self-efficacy to avoid indoor tanning (suggestions for how to have daughter refuse indoor tanning invitations), and interest in alternatives to indoor tanning (such as using sunless tanners or going with friends for spa treatments rather than indoor tanning). A key tenet was that the intervention needs to provide parents skills for communicating with their teens (ie, active listening, self-disclosure, showing empathy, and managing conflict), not just information on the risks of indoor tanning. From TT, a number of intervention posts contained links to news stories or stories provided by public health organizations from mothers and daughters about the risks of indoor tanning and their wish they had not given permission to indoor tan or avoided indoor tanning. These stories should be very effective at influencing individuals to alter their behaviors [51] because (1) people transported into a narrative world will alter their beliefs based on information, claims, or events depicted [52], (2) individuals identify with characters in a story, and identification increases the likelihood of social influence [53,54], and (3) narratives shift normative beliefs about risks [55-62]. To test our theoretical framework, all intervention messages are classified in 3 ways: (1) narrative versus didactic, (2) social norms–based versus not, and (3) appearance- versus health risk–based. Secondary analysis will probe which type of messages drive the most engagement among participants.

TT and diffusion of innovations theory (DIT) [63] were used to explain importance of soliciting user engagement from the mothers in the social media intervention, in the form of comments, shares, and likes. These theories guided our plan to encourage user-generated content and discussion on Facebook to capitalize on the interpersonal and interpretive processes in social networks that can produce sustained changes in health beliefs and behaviors. For instance, in social media, user-generated content such as testimonials and comments from other mothers, especially those phrased as stories, may be more powerful than conventional persuasive messages in posts alone, according to TT [51]. Likewise, DIT [63] explains how comments, shares, and likes from users in the social media should increase dissemination and impact of the intervention posts. It holds that social influence occurs through a process of delivering both carefully crafted messages and diffusion of these messages by community members, especially opinion leaders. The intervention will continually invite mothers to provide comments, shares, and likes in the hopes that opinion leaders will emerge in each of the Facebook groups. These opinion leaders should stimulate collective action among the mothers because people depend on them for information, especially about issues that carry risk and produce uncertainty [64-66], which opinion leaders deliver through their central position in a social group and links to outside information sources [63]. The information shared among mothers in the Facebook groups should breed collective action, as mothers interpret and respond to it through social comparison [63,67-69]. Mothers are expected to routinely compare themselves with other social network members [70] and conform with these peers to avoid uncertainty that arises when attitudes and behavior deviate [71]. In the process, they perceive themselves in abstract social categories and roles (eg, female, friend, mother, white, healthy person), which become part of their collective identity in the group, stabilizing behavior changes [67,68]. User engagement will be assessed in the form of number of posts, comments, likes, and views in the *Health Chat* program to test its influence on intervention outcomes.

#### Content

All participants will be invited to private Facebook groups to participate in the *Health Chat* program. The privacy setting in these groups is set to “secret” to prevent members and content of the group being visible to the public, including other Facebook users. Members of a private group with a “secret” privacy setting can only see information in each other’s profiles as indicated by their personal privacy settings. Members must also be invited to the group by the group administrator who will be a study staff member. The content of *Health Chat* is tailored to mothers and although only mothers will be in the Facebook groups, they will be encouraged to share content with their daughters. Posts will occur twice daily for 12 months for a total of 720 posts. Mothers will be encouraged to contribute their own content to the Facebook group via comments, original posts to share opinions or pose questions, and participation in group activities. Each group will be hosted by a community manager who will oversee the editorial calendar, maintain the feed, stimulate engagement, and monitor the broader media environment to discover trending topics and new research findings to post.

Our preliminary focus groups of mothers and key informant interviews of CSH coordinators revealed greater interest in a Facebook group focused broadly on health as opposed to a single topic like indoor tanning. For this reason, the *Health Chat* program will address health topics identified as of high interest by our focus group participants and CSH coordinators. These topics include healthy lifestyle, mental health, mother-daughter communication, and substance use. An advisory board of experts on these health topics provided evidence-based protocols and resources, which were then converted to Facebook posts by our team. In the *Health Chat* feed, 80% of posts were developed in advance based on evidence-based interventions and resources while 20% of posts will be pulled from emerging research and current events (eg, news reports of new tanning legislation, public service announcements about the health topic) relevant to the health topics.

#### Indoor Tanning Content

Indoor tanning content was developed by the investigators and a social media marketing expert using information from published literature on risk factors, evidence-based intervention content from published trials targeting indoor tanning [43,46,72-75], public health campaigns from major nonprofit organizations (eg, CDC, Skin Cancer Foundation, etc), and investigator-developed, video-recorded interviews of local mothers and professionals about the risks of indoor tanning, experiences with skin cancer, and mother-daughter communication role modeling.

Facebook posts on indoor tanning are intended to achieve the following: (1) increase awareness of state policy on indoor tanning by minors and teen interest in indoor tanning, (2) improve knowledge of indoor tanning risks, including skin damage (wrinkling/aging) and cancer, (3) teach mothers skills and improve self-efficacy for resisting daughters’ requests to indoor tan (eg, starting conversations, addressing sensitive topics, and managing conflict), (4) convey the importance of modeling tanning avoidance to daughters, (5) increase understanding of the reasons why adolescent girls indoor tan (eg, for stress reduction, self-medication of seasonal affect disorder, peer pressure, etc), (6) highlight behavioral alternatives to indoor tanning for adolescents (eg, sunless tanning, yoga, exercise, manicures/pedicures, and other spa treatments that enhance appearance, body image, and stress coping skills), (7) promote behavioral alternatives [72], and (8) give advice to avoid sun tanning and practice sun protection (ie, wear protective clothing, hat, and eyewear; seek shade; avoid midday sun; apply/reapply sunscreen with SPF 15+) grounded in SCT. Each message was designed according to our theoretical framework to ensure messages are balanced across (1) didactic versus narrative, (2) social norms based versus not, and (3) appearance- versus health risk-based messages. Once messages were developed, the entire investigative team reviewed the messages and made edits according to consensus. To evaluate the acceptability and readability of messages, focus groups were conducted with mothers of teenage daughters who viewed the messages in a private Facebook group for 1 week. Focus group participants rated each message on clarity, aesthetics, negative versus positive valence, interest, credibility, similarity to typical social media posts, and likelihood they “like,” comment, or share the post. Messages were then refined based on feedback.

### Control Condition

The East Tennessee State University Center for Prescription Drug Abuse/Misuse was consulted for content on opiate drug abuse. Relevant content from their website, as well as the Tennessee State Government [76], Kids Health [77], and National Institute on Drug Abuse for Teens websites [78], were converted into intervention posts.

### Measures

The primary outcomes are mothers’ permissiveness for daughters’ indoor tanning, mother and daughter indoor tanning behavior, and mothers’ support for stricter bans on indoor tannings in minors. Engagement with the *Health Chat* program and potential moderators and mediators of campaign effectiveness will also be assessed.

#### Primary Outcomes

Mothers’ permissiveness for daughters to tan indoors will be assessed using 4 Likert-type items (1=strongly disagree, 5=strongly agree) assessing permissiveness toward their teenage daughters’ indoor tanning [79]. Example items include, “I would allow my daughter to indoor tan,” and “I think it’s OK for my daughter to indoor tan” (Cronbach alpha=.97). Daughters will be asked the same 4 items to assess their perceptions of mothers’ permissiveness (Cronbach alpha=.95) [46]. Maternal permissiveness will be assessed at baseline and both follow-ups by the combined average ratings across the 6 items.

Indoor tanning behavior will be assessed by asking mothers and daughters to report on their indoor tanning over the last year using a single open-ended item (ie, “How many times in the past year have you used a tanning bed or booth?”) [80] Similar measures had strong positive correlations with diary measures of indoor tanning behavior (*r*=.77-.86, *P*<.001) in previous work [81,82]. Intention to indoor tan will also be assessed (ie, How likely is it that you will indoor tan in the next 3/6/12 months; 7-point Likert response scale), along with intention to get a sunless tan (eg, self-tanners, spray tans) in the next 12 months. We also will include an item specific to the months of December-March to capture indoor tanning during the seasonally high months of indoor tanning use. Indoor tanning behavior and intentions will be assessed at baseline and both follow-ups.

Support for strengthening bans on indoor tanning by minors will be measured via the Web server, which will record whether mothers who click on the link to “sign” the petition to strengthen the ban on indoor tanning and forward it to their legislator. At the final follow-up, mothers will be asked how much they support bans on indoor tanning by minors and about their reasons for either signing or not signing the petition.

#### Other Health Behaviors

Nineteen questions were included in the surveys to assess the other health behaviors addressed in the social media program. Participants rated their overall health status as excellent to poor. They described their diet by reporting the number of servings of fruits and of vegetables eaten each day and the number of times they drank regular soda or pop that contained sugar or sugar sweetened drinks (not 100% fruit juice or diet/artificially sweetened) in the past 30 days. Body mass index was calculated by asking for height (in inches) and weight (in pounds). Participants also described their regular physical activity, indicating how many times they engaged in vigorous and in light or moderate activities for at least 10 minutes per week. Alcoholic beverage intake was assessed by both number of days consuming at least 1 alcoholic drink in the past 30 days and number of times 4 or more alcoholic drinks were consumed in a row (binging) in the past 2 weeks was reported, along with smoking behavior (ie, smoking history; smoked at least 100 cigarettes in their lifetime) and current smoking (currently smoke every day, some days, or not at all). Mental health was assessed by asking how many days in the past 30 days was their mental health not good and disability was measured as the number of days in the past 30 days when poor physical or mental health kept them from doing their usual activities. Compliance with human papillomavirus (HPV) vaccination advice was assessed by asking if the daughter had been vaccinated and if so how many shots she received. Finally, 2 items measured abuse of prescription drugs: have you ever or in the past 6 months used a drug that was not prescribed for you or that you took only for the experience or feeling it caused even once? 

#### Engagement

Mothers’ engagement (ie, number of posts, comments, likes, and views with the *Health Chat* program) will be extracted from the Facebook page using a computer program.

Maternal communication will be assessed using 8 items asking mothers if they have talked to their daughters about indoor tanning in the past year. For example, “Within the past year, I have talked with my daughter about the importance of not being pressured to go to the tanning bed to fit in.” Response options will include “yes,” “no,” and “I prefer not to answer.” Mother-daughter relationship quality will be assessed using 2 Likert-type items (1=strongly disagree, 5=strongly agree): “I let my daughter make her own decisions,” and “Overall, I am satisfied with the way my daughter and I communicate.” Daughters will be asked the same 2 items to assess their perceptions of relationship quality. At the end of intervention follow-up (1-year), mothers will also be asked about how they shared information from the Facebook group with their daughter (eg, showed daughter a post in the private group).

### Analysis Plan

Hypotheses will be tested using a multilevel (mother-daughter dyad nested in Facebook private group) structural equation model (SEM). The following specific tests within the multilevel SEM will be used to evaluate the primary hypotheses in order to examine the effects of the social media campaign on indoor tanning outcomes. For the hypothesis regarding mothers’ permissiveness for daughters to indoor tan, mothers’ permissiveness (a level 1 variable) will be specified as a multiple indicator latent construct and regressed on the treatment indicator (campaign with prescription drug messages (control) vs campaign with indoor tanning messages (intervention), a level 2 variable). Similarly, for the hypothesis regarding daughters’ perceptions of mothers’ permissiveness, daughters’ perceptions (a level 1 variable), a multiple indicator latent construct, will be regressed on the treatment indicator (a level 2 variable). Mother and daughter perceptions will be correlated. In testing the hypothesis relating to indoor tanning frequency, mothers’ and daughters’ tanning (level 1 variables) will be specified as a count of the number of tanning sessions (in the past 3 months) and regressed on the treatment indicators (a level 2 variable), using a zero-inflated negative binomial distribution (which simultaneously models the effect of the intervention on the prevalence and frequency (among tanners) of tanning sessions). Mother and daughter behavior will be correlated. Finally, the hypothesis relating to mothers’ support for IT bans will be tested by regressing mothers’ signatures on the Web-based petitions (a level 1 binary variable—signed or not signed) on the treatment indicator (a level 2 variable) in a multilevel SEM.

#### Moderators

Differential effects of treatment on the outcomes associated with characteristics of the mothers and daughters (ie, demographics, political ideology, skin cancer history, and skin phenotype [83]) and their relationship (maternal communication and relationship quality [84-86]) will be tested using multiple group SEM (for categorical characteristics) and a treatment by characteristic interaction term (for continuous characteristics). All moderators will be level 1 variables. Tests of moderation will be built on top of the multilevel models with latent variables described above. A Holm-Bonferroni correction will be applied to adjust for multiple exploratory tests. These effects, tested in secondary analyses, will need to be large in order for a significant effect to be detected.

#### Mediators

Theorized mediators (ie, indoor tanning intentions, attitudes toward indoor tanning [73,87-89], conditional perceived susceptibility to skin damage [90], self-efficacy to resistant indoor requests, and mother-daughter indoor tanning–specific communication) and campaign engagement (level 1 variables) from the 6-month follow-up will be regressed on the treatment indicator (a level 2 variable) in a multilevel SEM. If treatment effects on mediators emerge, a full multilevel SEM with direct and indirect effects on the primary outcomes of IT permissiveness and behavior will be assessed using a causal mediation framework [91,92]. A bootstrap resampling procedure will be used to construct 95% confidence intervals around each indirect effect estimate [93,94].

#### Seasonality Issues

In order to account for the issue of seasonality (ie, that indoor tanning is more common during certain times of the year), we will measure indoor tanning at each follow-up, asking specifically about tanning during the months of December-March. We will also include date of measurement as a covariate.  We will control for month of assessment and number of months that have elapsed since baseline. In addition, the treatment and control group, randomized together, will be surveyed at precisely the same times to ensure equivalency.

## Results

The first wave of the intervention began in September 2016. We anticipate on continuing recruitment through October 2018 and completing this study by October 2019. Results will be examined at that time.

## Discussion

### Mothers as a Key Intervention Target

The proposed research will fill 2 gaps in the existing literature by (1) decreasing mothers’ permissiveness to allow their daughters to tan so as to maximize public policy on indoor tanning, and (2) using social media to deliver a health communication campaign targeting mothers. Past research on indoor tanning policy has examined industry compliance and policy impact on mothers and daughters [29,30,95-109], but has not evaluated health communication interventions to maximize the impact of indoor tanning policy. Studies of policy interventions on sun safety of youth in general are rare, limited to a few studies on policy adoption by US and Australian schools [110,111] (including a successful intervention by our team [112]) and recreation centers [113,114].

### Advantages of Social Media as Intervention Modality

Social media has revolutionized communications and offers several advantages for an indoor tanning campaign. Social media can reach many across the United States, including mothers [115], because most US adults use the Internet and social media [115]. Use is especially high by women (72% are on Facebook, 25% on Pinterest, 16% on Instagram, and 15% on Twitter) [115]. Further, most US adults (80%) use the Internet to retrieve health information [116] because it is low-cost, available 24/7, private [117], can be personalized, and enhances social connections [118]. At least 20% of women aged 25-44 use social media to post about their health and share health videos/images [119-121]. Social media users create and share content that provides opportunity for information dissemination, social norm change, and broad impact [122,123]. Health is a popular topic on social media as indicated by the formation of patient communities and health-related hashtags [119-122,124]. A 2011 survey found that 34% of Internet users had read a commentary or experience about health/medical issues on a website or blog [125]. Social media can also stimulate collective action [112,126]. Social media has been at the forefront of large collective political actions, including oppositional movements in Egypt, Occupy Wall Street, the Tea Party [127], and the Obama presidential campaign (which had 32 million Facebook friends, 22 million Twitter followers [128], and 300 million YouTube views [129], and digitally raised US $525 million) [127]. Social media can also heighten awareness, frame issues, develop/expand networks, and motivate Web-based and offline collective actions (eg, writing letters, organizing meet-ups, attending hearings/events, registering to vote, and sharing information [112,126,130-132]). For example, an organ donor registration effort by Facebook in 2012, yielded 13,054 new registrations in its first day (21.1 times more than an average day) [133]. The proposed indoor tanning social media campaign is intended to change attitudes about indoor tanning and ultimately, elevate support for stricter, more effective bans on minors’ access to indoor tanning facilities. Currently, very few studies have been published on social media in public health campaigns, so the proposed project will also fulfill calls by National Institutes of Health for research to identify best practices for using social media and Web 2.0 technologies in health behavior interventions. Social media interventions are not without limitations. One challenge is that frequency of social media use varies across individuals with some people logging in several times per day and others logging in once per week or less. Nonusers and infrequent users may be less likely to benefit from social media–delivered interventions, unless they are convinced to engage. In the present study, we will conduct a year-long campaign of twice daily posts to provide numerous opportunities for participants to see the content. We will also employ social media marketing strategies to encourage engagement. We will also study engagement patterns by user characteristics to inform the nascent, but much needed literature on engagement in social media interventions [134].

Many federal and state agencies, nonprofits, and health care providers already use social media extensively to disseminate information [135,136] (eg, National Institute on Drug Abuse, CDC, and Environmental Protection Agency), and also use generated video contests to reach young people on immunization [137], tobacco [138], organ donation [139], and HPV vaccine [137,140]. The indoor tanning industry also actively markets its services on social media [141]. Thus, the results of this trial will deliver social media content grounded in theory, and test it in a randomized design with state-of-the-art measures. Also, they will contribute much needed insights on how to employ social media for health behavior change and disease prevention both for indoor tanning and other health risk behaviors and inform future social media efforts by public health and health care organizations.

## Appendix

Multimedia Appendix 1 Summary sheets from grant review.

## Abbreviations

CDC: Centers for Disease Control
FDA: Food and Drug Administration
CSH: coordinated school health
DIT: diffusion of innovations theory
HPV: human papillomavirus
SCT: social cognitive theory
SEM: structural equation model
TT: transportation theory
