# Supplementary material for: Engaging Moms on Teen Indoor Tanning Through Social Media: Protocol of a Randomized Controlled Trial
Source: JMIR Res Protoc. 2016 Nov 29;5(4):e228. doi: 10.2196/resprot.6624 (PMC5147712; doi:10.2196/resprot.6624)
Supplement: Multimedia Appendix 1 [file resprot_v5i4e228_app1.PDF]

SUMMARY STATEMENT
(Privileged Communication)

PROGRAM CONTACT:
Wen-Ying Chou
(240)276-6954
chouws@mail.nih.gov

Release Date: 06/17/2014

Application Number: 1 R01 CA192652-01

Principal Investigators (Listed Alphabetically):
BULLER, DAVID B PHD (Contact)
PAGOTO, SHERRY L. PHD

Applicant Organization: KLEIN BUENDEL, INC.

Review Group: PRDP
Psychosocial Risk and Disease Prevention Study Section

Meeting Date: 06/02/2014
Council: OCT 2014
Requested Start: 12/01/2014

RFA/PA: PA13-302
PCC: W3CI
Dual IC(s): HD, AA, DA, HL

Project Title: Likes Pins and Views: Engaging Moms on Teen Indoor Tanning Thru Social Media

SRG Action: Impact Score: 20 Percentile: 12
Next Steps: Visit http://grants.nih.gov/grants/next_steps.htm
Human Subjects: 30-Human subjects involved - Certified, no SRG concerns
Animal Subjects: 10-No live vertebrate animals involved for competing appl.
Gender: 2A-Only women, scientifically acceptable
Minority: 1A-Minorities and non-minorities, scientifically acceptable
Children: 1A-Both Children and Adults, scientifically acceptable
Clinical Research - not NIH-defined Phase III Trial

Project Year	Direct Costs Requested	Estimated Total Cost
1	499,985	819,306
2	499,988	819,310
3	499,987	819,309
4	499,989	819,312
5	496,961	814,350
TOTAL	2,496,910	4,091,587

ADMINISTRATIVE BUDGET NOTE: The budget shown is the requested budget and has not been adjusted to reflect any recommendations made by reviewers. If an award is planned, the costs will be calculated by Institute grants management staff based on the recommendations outlined below in the COMMITTEE BUDGET RECOMMENDATIONS section.

1R01CA192652-01 BULLER, DAVID

COMMITTEE BUDGET RECOMMENDATIONS

RESUME AND SUMMARY OF DISCUSSION: This application seeks support to evaluate the effectiveness of a social media public health communication campaign to reduce indoor tanning by engaging the mothers of adolescent girls. This compelling work offers high impact and concerns the public health challenge of melanoma. Reviewers noted many strengths in this work: the excellent significance of the topic and potential impact on statewide policy about indoor tanning; the outstanding investigators and research environment; a novel intervention focus on the mothers and incorporation of social media both considered exceptional by the reviewers; an excellent approach which included a randomized controlled trial; inclusion of excellent mediator and moderator variables; and a strong analytic plan. Reviewers voiced a few concerns: qualitative aspects of this work such as the focus groups are not well integrated; concern that participants will not follow through; potential problems with loss to follow up as well as minority recruitment; the intervention does not account for seasonal impact on recruitment and analysis; the incentives to complete the survey are possibly insufficient; and an overly staffed and geographically complex administrative structure might limit the study feasibility and implementation. Overall, reviewers opined high enthusiasm for this outstanding application with notable strengths and few weaknesses.

DESCRIPTION (provided by applicant): Social media has dramatically changed both the Internet landscape and how we interact with one another. Users gather information and serve as active sources of information, not just passive recipients, influencing who receives information and its credibility. Consequently, many Americans are much more engaged in public discourse through social media than they were when older media such as television dominated the communication terrain. Our team proposes to evaluate the effectiveness of employing social media in a public health communication campaign directed to mothers of adolescent daughters with the goal of decreasing indoor tanning by adolescent girls. This research is significant because many health agencies and organizations rely on social media yet there is a paucity of research on the potential influence of social media in health communication campaigns. This research gap has led to calls for investigations that identify best practices for social media in such campaigns, including by NIH, and our proposed research will fill this gap. The focus on preventing indoor tanning by minors is also significant because indoor tanning has been linked to elevated risk for developing skin cancer. Many states have enacted bans on access by minor but many include exceptions for parental permission, so if our campaign reduces mothers' permissiveness, it should reduce indoor tanning in that circumstance. The specific aims are to: 1) develop and implement a social media campaign for mothers on health and wellness of adolescent daughters that includes theory-based messaging advocating adolescent girls avoid indoor tanning and 2) evaluate the effectiveness of the indoor tanning messages at a) decreasing mothers' reported permissiveness for daughters to indoor tan; b) decreasing daughters' perception of mothers' permissiveness; c) decreasing the prevalence of indoor tanning by mothers and daughters; and d) increasing the number of mothers who support strengthening bans on indoor tanning by minors. Mother-daughter pairs from secondary schools, recruited with the help of the Coordinated School Health program, will be enrolled in a group-randomized controlled design; Tennessee requires parental permission for minors to indoor tan. The indoor tanning messages will be delivered to mothers through an integrated social media campaign using Facebook, Twitter, YouTube, Pinterest, and Instagram over two school years (18 months). Chapters will be assigned to receive a health and wellness social media campaign with (intervention) or without (control) indoor tanning messages. Mothers and daughters will be assessed at baseline and followed up at 6- and 12-months after the campaign concludes with validated self-report measures of mothers' permissiveness for daughter to indoor tan and mothers' and daughters' prevalence of indoor tanning. Mothers' support for indoor tanning bans will be objectively assessed by asking them to sign online petitions to school principals and state officials. Secondary

aims are to analyze the moderating effects of characteristics of mothers and daughters (e.g., desire to tan) and mediation of campaign effectiveness by theoretical concepts and campaign engagement.

PUBLIC HEALTH RELEVANCE: Indoor tanning (IT) increases the risk of melanoma and 34 states have responded by passing bans on access to IT facilities by minors to reduce the rates of melanoma. A social media campaign will be delivered to mothers with adolescent daughters designed to convince them not to allow their daughters to indoor tan. Reducing parental consent will improve the effectiveness of IT restrictions and help reduce melanoma in the United States.

CRITIQUE 1:

Significance: 2
Investigator(s): 2
Innovation: 1
Approach: 2
Environment: 3

Overall Impact: The proposed study is exceedingly significant and extremely innovative. The multi-disciplinary team is strong. One concern is with regard to whether the primary results are appropriate to justify a study of this scope, the feasibility of the team carrying out the study, given that they are spread out across various parts of the country (although data collection to be led by a co-investigator in Colorado and data collection to occur in Tennessee) and some questions that need to be resolved in the approach. Overall, this works offers a potential high impact.

1. Significance:

Strengths

- Preventing skin cancer, especially melanoma by reducing indoor tanning among mother-adolescent dyads is a significant topic area.
- Significance is enhanced by the investigators proposing to intervene on indoor tanning behaviors of both the mothers and daughters through use of a mother-daughter dyadic model.

Weaknesses

- The preliminary studies (presented in boxes 1-5) do not specifically support the aims to develop and evaluate a social marketing campaign on indoor tanning. Although the investigators present some of their preliminary work in evaluating other behaviors related to social media (e.g., weight loss and exercise), these conditions and behaviors might lend themselves to a positive social media approach more than a behavior such as indoor tanning. In addition, a lot of space is taken up reporting preliminary results, which in some cases do not appear to be directly related to the current application.

2. Investigator(s):

Strengths

- Very strong multi-disciplinary team with expertise in indoor tanning, social media, and theory-based intervention work.

Weaknesses

- Although this is a social media study through use of Internet, there are still concerns that the investigators (both multiple PIs and co-investigators) are spread out across the country). For

instance, Dr. Buller who is in Colorado will oversee data collection which will take place in East Tennessee. The co-investigator (Dr. Pagoto is in Massachusetts) and other co-investigators are in the “data collection” site of East Tennessee.

- This project appears to be overstaffed, especially with senior-level investigators and consultants. Although the multiple PI plan is justified, it is unclear why all of the various co-investigators, staff, and consultants are needed to carry out this project (e.g., statistical personnel are included as well as a senior statistical consultant) and there is potential overlap in activities of the multiple co-investigators and staff. This requires further clarification.

3. Innovation:

Strengths

- Mother-daughter dyadic model to address decreasing indoor tanning through use of social media and incorporating public policy perspective of promoting banning of indoor tanning among minors is extremely innovative.

Weaknesses

- None noted.

4. Approach:

Strengths

- Very informative well-developed graphic illustration of the proposed social media campaign.
- Excellent and appropriate design: group-randomized controlled design with assessments at baseline, and 6- and 12-month post-assessment.
- Content and messaging is well constructed and sufficiently described.
- The assessments (e.g. measures, detail, psychometrics, appropriateness, inclusion of potential mediators) are strong.
- The hypotheses and accompanying statically analysis section is organized and comprehensive.

Weaknesses

- The qualitative component (e.g., 5 focus groups) was not well incorporated into the rest of the study design and analyses. In addition, there is a discrepancy in the narrative and budget sections on incentive amount (in narrative, it is listed as $50 and in budget; it is listed as $40).
- Some concern as to whether or not the investigators will get the participants to complete surveys since the incentive amount is extremely low ($3), especially since they will be conducted online. Although the assessments are well detailed, the investigators did not state how long they anticipate the surveys will take to complete (again drawing concerns with regard to completion of these surveys, especially at 6- and 12- months post-test). It would be disappointing to conduct such a well-designed intervention and potentially not get the outcome information needed at post-test.

5. Environment:

Strengths

- Although all of the various environments (respective institutions in Colorado, Tennessee, Massachusetts) are all extremely strong and supportive, as mentioned above, there are concerns with regard to cohesiveness and feasibility of this study being carried out, especially

since data collection is proposed to be led by Dr. Buller in Colorado but will take place in Tennessee.

Weaknesses

- None noted.

Protections for Human Subjects:

Acceptable Risks and/or Adequate Protections

Data and Safety Monitoring Plan (Applicable for Clinical Trials Only):

Acceptable

Inclusion of Women, Minorities and Children:

G2A - Only Women, Acceptable

M1A - Minority and Non-minority, Acceptable

C1A - Children and Adults, Acceptable

Vertebrate Animals:

Not Applicable (No Vertebrate Animals)

Biohazards:

Not Applicable (No Biohazards)

Budget and Period of Support:

Recommended budget modifications or possible overlap identified:

- The amount of time allocated to multiple PIs, co-investigators, consultants, and staff (e.g., coordinators, managers, research associates, etc.) appears to be very high and there may be an overlap in both expertise and actual responsibilities on the project.

CRITIQUE 2:

Significance: 1
Investigator(s): 1
Innovation: 2
Approach: 2
Environment: 1

Overall Impact: The proposed study will examine the efficacy of a social media campaign to reduce indoor tanning by minors and support for indoor tanning among mothers. Goal is to reduce permissiveness of mothers, and tanning behavior while increasing support for legislation limiting or banning indoor tanning. Team has experience in many parallel areas but the use of social media for this outcome is new. Willingness to sign a petition for legislation may have little correlation with actual support. Methods show attention to detail such as loss to follow-up and contamination. This work offers high potential to impact reduction of indoor tanning among minors.

1. Significance:

Strengths

- Little is known about how social media influences health communication
- Maternal permissiveness and behavior are predictors of daughter indoor tanning but few interventions have targeted mothers' behavior.
- Information shared by friends on social media may have more credibility than that of professionals
- Social media use is high among women in the age range typical of women who are mothers of teenagers

Weaknesses

- None noted.

2. Investigator(s):

Strengths

- Principal Investigator has track record of skin cancer prevention research
- Principal Investigator is experienced in technology based interventions
- Co-investigators are outstanding and provide complementary support to Principal Investigator's expertise

Weaknesses

- None noted.

3. Innovation:

Strengths

- Campaigns to target mother permissiveness on daughters' behavior have not been tested for indoor tanning
- Use of social media exclusively to deliver intervention content
- Few studies have evaluated use of social media in public health campaigns
- First study to examine whether a social media campaign can increase support for a public health policy change

Weaknesses

- Interventions targeting mothers to improve health of daughters is not unique

4. Approach:

Strengths

- Integration of evidence based theories since no existing theory for social media communication exists
- Power calculations appropriately account for loss to follow up and inflate for minority recruitment and participation in indoor tanning.
- Well-articulated plan to limit contamination

Weaknesses

- There are no testable hypotheses stated
- Support for government regulation may not align with individual preferences and decisions and ignores the current political landscape
- Tanning may have a seasonal effect which is not accounted for in recruitment or analysis
- In the pilot, while most would sign a petition, few supported permission laws or bans. This suggests that support for petitions is already high and the intervention may face a ceiling effect. It also suggests that signing a petition is a poor proxy endpoint for support of legislation limiting or banning access.
- Increasing concerns about privacy in social media. How will mothers concerns about exposing self/daughter through the various posting requests be managed?

5. Environment:

Strengths

- Facilities are outstanding for the support of research
- Infrastructure at KB to support multisite studies is excellent
- Data management at KB team is experienced
- Attention to detail in describing the technology and security infrastructure at KB is commendable.

Weaknesses

- None noted.

Protections for Human Subjects:

Acceptable Risks and/or Adequate Protections

- Planned enrollment N does not match N listed in human subjects section.

Data and Safety Monitoring Plan (Applicable for Clinical Trials Only):

Acceptable

Inclusion of Women, Minorities and Children:

G2A - Only Women, Acceptable

M1A - Minority and Non-minority, Acceptable

C1A - Children and Adults, Acceptable

Vertebrate Animals:

Not Applicable (No Vertebrate Animals)

Biohazards:

Not Applicable (No Biohazards)

Resource Sharing Plans:

Acceptable

Budget and Period of Support:

Recommend as Requested

CRITIQUE 3:

Significance: 4
Investigator(s): 1
Innovation: 1
Approach: 3
Environment: 1

Overall Impact: This study proposes to reduce indoor tanning by adolescent girls by evaluating the effectiveness of a social media public health communication campaign directed at their mothers. Mothers of high school students in Eastern Tennessee will be targeted for this public health campaign with randomization by county in collaboration with the secondary school systems. The public health campaign will focus on many health behaviors somewhat hiding the true purpose of the study, which is the reduction of indoor tanning. This application is extremely compelling and well written, led by a highly qualified team, and built based on a very strong theoretical framework. The use of social media to affect change is innovative, and this innovation is further enhanced by the outcome of parental letters to policy makers, which is included as a study outcome. The enthusiasm for this excellent application is only mildly limited by the mostly subjective outcome measures, the large expense required to build a “stealth” intervention, and the unjustified sole focus on mothers and daughters.

1. Significance:

Strengths

- Indoor tanning is associated with melanoma, which is an increasing cause of morbidity and mortality.

Weaknesses

- 34 of 50 states are addressing indoor tanning via policy methods, though the application does make clear that policy implementation has been challenging.
- The application does not clearly demonstrate the extent to which adolescent indoor tanning is contributing to the rise in melanoma.

2. Investigator(s):

Strengths

- This is a uniquely qualified team to address the issues related to indoor tanning among adolescents through social media.
- The investigators have very complimentary experience.
- The multiple PI plan has good detail.

- There is a well-developed external advisory board for the rest of the wellness education in the social media campaign.

Weaknesses

- None noted.

3. Innovation:

Strengths

- Using social media to advance a public health campaign is highly innovative.
- Attempting to influence policy makers through a clinical research study is admirable and ambitious, but justified.

Weaknesses

- None noted.

4. Approach:

Strengths

- Both the intervention components and its dissemination make masterful use of a theoretical framework for the research in the very well-written significance section.
- It is very clever to make the indoor tanning intervention part of a larger social media campaign, making it more likely to engage mothers that allow their daughters to tan.

Weaknesses

- The application gives good reasons why Tennessee is a good state to perform this intervention, but the fact that a policy already exists in this state makes this reviewer wonder whether another state with high rates of indoor tanning that does not have legislation exists. The details on the 16 states without policy are not given in terms of population, indoor tanning use among teens, etc.
- The design benefits from having a large social media campaign with the indoor tanning component being only a part, but this creates quite an expensive program that has a smaller percentage dedicated to the behavior of interest.
- The main outcomes measures are all survey-based, and it would be wonderful if additional objective outcomes could be identified.
- The application does not clearly state why male adolescents as well as fathers are not targets of this study.

5. Environment:

Strengths

- The Principal Investigators have been highly supported in the past to conduct their research suggesting an excellent environment.

Weaknesses

- None noted.

Protections for Human Subjects:

Acceptable Risks and/or Adequate Protections

Data and Safety Monitoring Plan (Applicable for Clinical Trials Only):

Acceptable

Inclusion of Women, Minorities and Children:

G2A – Only Women, Acceptable

M1A – Minority and Non-minority, Acceptable

C1A – Children and Adults, Acceptable

Vertebrate Animals:

Not Applicable (No Vertebrate Animals)

Biohazards:

Not Applicable (No Biohazards)

Budget and Period of Support:

Recommend as Requested.

THE FOLLOWING RESUME SECTIONS WERE PREPARED BY THE SCIENTIFIC REVIEW OFFICER TO SUMMARIZE THE OUTCOME OF DISCUSSIONS OF THE REVIEW COMMITTEE ON THE FOLLOWING ISSUES:

PROTECTION OF HUMAN SUBJECTS (Resume): ACCEPTABLE. There are no concerns.

INCLUSION OF WOMEN PLAN (Resume): ACCEPTABLE. The study sample will be comprised of mother-daughter dyads.

INCLUSION OF MINORITIES PLAN (Resume): ACCEPTABLE. All race and ethnic groups will be targeted and enrolled in this study.

INCLUSION OF CHILDREN PLAN (Resume): ACCEPTABLE. Adolescent children aged 14 to 17 and their adult mothers will be enrolled in this study.

COMMITTEE BUDGET RECOMMENDATIONS: Reviewers note overstaffing by senior investigators with overlapping roles.

NIH has modified its policy regarding the receipt of resubmissions (amended applications). See Guide Notice NOT-OD-10-080 at http://grants.nih.gov/grants/guide/notice-files/NOT-OD-10-080.html.
The impact/priority score is calculated after discussion of an application by averaging the overall scores (1-9) given by all voting reviewers on the committee and multiplying

by 10. The criterion scores are submitted prior to the meeting by the individual reviewers assigned to an application, and are not discussed specifically at the review meeting or calculated into the overall impact score. Some applications also receive a percentile ranking. For details on the review process, see http://grants.nih.gov/grants/peer_review_process.htm#scoring.

MEETING ROSTER

Psychosocial Risk and Disease Prevention Study Section
Risk, Prevention and Health Behavior Integrated Review Group
CENTER FOR SCIENTIFIC REVIEW
PRDP
June 02, 2014 - June 03, 2014

CHAIRPERSON

EPSTEIN, LEONARD H, PHD
SUNY DISTINGUISHED PROFESSOR
DEPARTMENT OF PEDIATRICS
DIVISION OF BEHAVIORAL MEDICINE
STATE UNIVERSITY OF NEW YORK AT BUFFALO
BUFFALO, NY 14214

MEMBERS

ABRANTES, ANA M, PHD
ASSOCIATE PROFESSOR
DEPARTMENT OF PSYCHIATRY AND HUMAN BEHAVIOR
ALPERT MEDICAL SCHOOL
BUTLER HOSPITAL
BROWN UNIVERSITY
PROVIDENCE, RI 02906

AYALA, GUADALUPE X, MPH, PHD
PROFESSOR
INSTITUTE FOR BEHAVIORAL
AND COMMUNITY HEALTH
SAN DIEGO STATE UNIVERSITY
SAN DIEGO, CA 92123

BASKIN, MONICA L, PHD
PROFESSOR
DIVISION OF PREVENTIVE MEDICINE
UNIVERSITY OF ALABAMA AT BIRMINGHAM
BIRMINGHAM, AL 35294

BEETS, MICHAEL W, PHD *
ASSOCIATE PROFESSOR
DEPARTMENT OF EXERCISE SCIENCE
ARNOLD SCHOOL OF PUBLIC HEALTH
UNIVERSITY OF SOUTH CAROLINA
COLUMBIA, SC 29208

BEFORT, CHRISTIE , PHD *
ASSOCIATE PROFESSOR
CO-DIRECTOR, BREAST CANCER SURVIVORSHIP
CENTER
DEPARTMENT OF PREVENTIVE MEDICINE
AND PUBLIC HEALTH
UNIVERSITY OF KANSAS MEDICAL CENTER
KANSAS CITY, KS 66160

BENNETT, GARY G, PHD
PROFESSOR OF PSYCHOLOGY
DEPARTMENT OF GLOBAL HEALTH AND MEDICINE
DUKE UNIVERSITY
DURHAM, NC 27705

BOUTELLE, KERRI N, PHD
PROFESSOR
DEPARTMENT OF PEDIATRICS
AND PSYCHIATRY
UNIVERSITY OF CALIFORNIA, SAN DIEGO
LA JOLLA, CA 92037

COUPS, ELLIOT J, PHD
ASSOCIATE PROFESSOR
DEPARTMENT OF MEDICINE
RUTGERS, THE STATE UNIVERSITY OF NEW JERSEY
ROBERT WOOD JOHNSON MEDICAL SCHOOL
NEW BRUNSWICK, NJ 08901

DAVISON, KIRSTEN , PHD
ASSOCIATE PROFESSOR
DEPARTMENT OF NUTRITION
HARVARD SCHOOL OF PUBLIC HEALTH
BOSTON, MA 02115

FOCHT, BRIAN CARL, PHD *
ASSOCIATE PROFESSOR
KINESIOLOGY, DEPARTMENT OF HUMAN SIENCES
COMPREHENSIVE CANCER CENTER
AND SOLOVE RESEARCH INSTITUTE
THE OHIO STATE UNIVERSITY
COLUMBUS, OH 43210

LEAHEY, TRICIA M, PHD
ASSISTANT PROFESSOR
WEIGHT CONTROL AND DIABETES
RESEARCH CENTER
MIRIAM HOSPITAL
BROWN MEDICAL SCHOOL
PROVIDENCE, RI 02903

LEVINE, MICHELE D, PHD
ASSOCIATE PROFESSOR
DEPARTMENT OF PSYCHIATRY
UNIVERSITY OF PITTSBURGH
PITTSBURGH, PA 15213

PAUL, IAN M, MD *
PROFESSOR
DEPARTMENT OF PEDIATRICS
COLLEGE OF MEDICINE
PENNSYLVANIA STATE UNIVERSITY
HERSHEY, PA 17033

POLLAK, KATHRYN I, PHD
ASSOCIATE PROFESSOR
DUKE CANCER CONTROL
AND POPULATION SCIENCES
DUKE UNIVERSITY MEDICAL CENTER
DURHAM, NC 27705

QUARELLS, RAKALE COLLINS, PHD
ASSOCIATE PROFESSOR
CARDIOVASCULAR RESEARCH INSTITUTE
MOREHOUSE SCHOOL OF MEDICINE
ATLANTA, GA 30310

SEVICK, MARY A, SCD, BSN *
PROFESSOR OF MEDICINE
DEPARTMENT OF POPULATION HEALTH
SCHOOL OF MEDICINE
NEW YORK UNIVERSITY
NEW YORK, NY 10016

STAIANO, AMANDA E, PHD *
ASSISTANT PROFESSOR
LABORATORY OF PEDIATRIC
OBESITY AND HEALTH BEHAVIOR
PENNINGTON BIOMEDICAL RESEARCH CENTER
LOUSIANA STATE UNVERSITY
BATON ROUGE , LA 70808

TATE, DEBORAH F, PHD
ASSOCIATE PROFESSOR
DEPARTMENT OF HEALTH
BEHAVIOR AND NUTRITION
UNIVERSITY OF NORTH CAROLINA
CHAPEL HILL, NC 27599

THOMPSON, DEBORAH I, PHD
ASSOCIATE PROFESSOR OF PEDIATRICS
USDA/ARS SCIENTIST/NUTRITIONIST-CHILD OBESITY
CHILDREN'S NUTRITION RESEARCH CENTER
BAYLOR COLLEGE OF MEDICINE
HOUSTON, TX 77030

WOLIN, KATHLEEN Y, SCD *
ASSOCIATE PROFESSOR
DEPARTMENTS OF PUBLIC HEALTH
SCIENCES AND SURGERY
STRITCH SCHOOL OF MEDICINE
LOYOLA UNIVERSITY CHICAGO
MAYWOOD, IL 60153

YAROCH, AMY L, PHD
EXECUTIVE DIRECTOR
GRETCHEN SWANSON CENTER
FOR NUTRITION
OMAHA, NE 68114

MAIL REVIEWER(S)

SCHUR, ELLEN A, MD
ASSISTANT PROFESSOR
DIVISION OF INTERNAL MEDICINE
DEPARTMENT OF MEDICINE
UNIVERSITY OF WASHINGTON
SEATTLE, WA 98104

SMITH, MICHAEL T, PHD
PROFESSOR
DEPARTMENT OF PSYCHIATRY, AND BEHAVIORAL
SLEEP
MEDICINE PROGRAM, CENTER FOR BEHAVIOR
AND HEALTH, SCHOOL OF MEDICINE
BAYVIEW MEDICAL CENTER JOHNS HOPKINS
UNIVERSITY
BALTIMORE, MD 21224

SCIENTIFIC REVIEW ADMINISTRATOR

FITZSIMMONS, STACEY , MPH, PHD
SCIENTIFIC REVIEW OFFICER
CENTER FOR SCIENTIFIC REVIEW
NATIONAL INSTITUTES OF HEALTH
BETHESDA, MD 20892

GRANTS TECHNICAL ASSISTANT

JENNINGS, CHRISOULA , BS
LEAD EXTRAMURAL SUPPORT
CENTER FOR SCIENTIFIC REVIEW
NATIONAL INSTITUTES OF HEALTH
BETHESDA, MD 20892

* Temporary Member. For grant applications, temporary members may participate in the entire meeting or may review only selected applications as needed.

Consultants are required to absent themselves from the room during the review of any application if their presence would constitute or appear to constitute a conflict of interest.
